# Traditional Japanese Kampo Medicine: Clinical Research between Modernity and Traditional Medicine—The State of Research and Methodological Suggestions for the Future

**DOI:** 10.1093/ecam/neq067

**Published:** 2011-02-17

**Authors:** Kenji Watanabe, Keiko Matsuura, Pengfei Gao, Lydia Hottenbacher, Hideaki Tokunaga, Ko Nishimura, Yoshihiro Imazu, Heidrun Reissenweber, Claudia M. Witt

**Affiliations:** ^1^Center for Kampo Medicine, Keio University School of Medicine, Shinjuku-ku, Tokyo, Japan; ^2^Institute for Social Medicine, Epidemiology and Health Economics, Charité University Medical Center, 10098 Berlin, Germany; ^3^Research Unit for Japanese Phytotherapy (Kampo), Department of Internal Medicine, University of Munich, Munich, Germany

## Abstract

The Japanese traditional herbal medicine, Kampo, has gradually reemerged and 148 different formulations (mainly herbal extracts) can be prescribed within the national health insurance system. The objective of this article is to introduce Kampo and to present information from previous clinical studies that tested Kampo formulae. In addition, suggestions on the design of future research will be stated. The literature search was based on a summary, up until January 2009, by the Japanese Society of Oriental Medicine and included only those trials which were also available in either Pubmed or ICHUSHI (Japan Medical Abstracts Society). We included 135 studies, half of these studies (*n* = 68) used a standard control and 28 a placebo control. Thirty-seven trials were published in English [all randomized controlled trials (RCTs)] and the remaining articles were in Japanese only. The sample size for most studies was small (two-third of the studies included less than 100 patients) and the overall methodological quality appeared to be low. None of the studies used Kampo diagnosis as the basis for the treatment. In order to evaluate Kampo as a whole treatment system, certain aspects should be taken into account while designing studies. RCTs are the appropriate study design to test efficacy or effectiveness; however, within the trial the treatment could be individualized according to the Kampo diagnosis. Kampo is a complex and individualized treatment with a long tradition, and it would be appropriate for further research on Kampo medicine to take this into account.

## 1. Background

### 1.1. Historical Background

Japanese traditional herbal medicine (Kampo medicine) obtained the unique features observed today during its phase of long historical development in Japan. In Japan, the administration of crude herbal drug formulations dates back by more than 1500 years. Recent decades have seen a revival of Kampo medicine in medical practice, accompanied by a scientific reevaluation and critical examination of its relevance in modern health care [1].

The term “Kampo”, which literally means “method from the Han period (206 BC to 220 AD) of ancient China”, refers to its origin from ancient China. The basic therapeutic handbook for the application of herbal prescriptions was the *Shang han lun*. During the Edo-period from 1600 onwards, the specific Japanese characteristics of Kampo took shape. The seclusion of Japan from the outside world led to ever increasing differences from the predominantly Chinese concepts. The huge variety of the thousands of Chinese crude drugs was reduced to *∼*300, those being the most efficacious drugs which were subsequently combined into *∼*300 prescriptions. From a pragmatic point of view, Japanese physicians criticized the highly theoretical and speculative nature of Chinese medicine as being inadequate to meet the problems of every-day practice. The strongest critique came from Yoshimasu Todo in the 18th century who wrote: “In clinical medicine, we should only rely on what we actually have observed by examination of the patient”. For Yoshimasu Todo, one way to gain data on the condition of the body was to examine the abdomen, for which he developed a refined palpation technique *(fukushin)* [2]. The results of the abdominal palpation should give additional clinical information in order to select the most appropriate herbal prescription for the patient. Yoshimasu Todo's pragmatic attitude and his abdominal palpation as a diagnostic procedure has had a strong influence on Kampo therapy right up until the present day [3].

It is not surprising that many Japanese physicians were drawn towards medical techniques from the West to improve their therapeutic options in surgery, but most of them continued to use traditional Kampo prescriptions for treating problems of internal medicine until the 19th century. At the end of the 19th century, it became obvious that for the urgent medical problems of that time, infectious diseases and acute surgical problems, Western medicine had better tools. The German system of medical education was adopted. In 1876, the government passed a regulation that all physicians were required to study Western medicine. The practice of Kampo was not forbidden but greatly inhibited and gradually declined [4]. However, after the Second World War, the first modern Kampo specialists carried on the traditions from the Edo-period. This revival of Kampo took place within a context dominated by modern Western medicine. The pragmatic and reductive approach of restricting Kampo therapy to clinically meaningful components helped to facilitate its gradual integration into modern medicine. Modern industrial society, in combination with longer life expectancy, has caused a shift in the predominant disease patterns, bringing to the therapeutic forefront chronic and degenerative diseases, functional and psychosomatic disorders and the multimorbidity of the elderly. These provide the main indications for the use of herbal drugs, not only with respect to treatment, but also for prevention [5].

Although rooted in Chinese tradition, Kampo medicine is not the same as modern traditional Chinese medicine (TCM). TCM emphasizes the traditional concepts of East Asian natural philosophy, such as Yin and Yang and the theory of the five elements. Japanese Kampo favors diagnostic methods that directly relate the symptoms to the therapy, bypassing speculative concepts. The vast array of crude drugs has been reduced in Kampo and also the quantity of each drug in the formulation is much lower. While Kampo still uses traditional prescriptions, TCM also tends to create new drug combinations [6].

### 1.2. Usage and Integration into Modern Medicine

Kampo traditional prescriptions have been included in the Japanese National Health Insurance drug list since 1971. A total of 148 Kampo herbal prescriptions are able to be funded to date. The application of Kampo has steadily increased and according to a survey by the Journal Nikkei Medical, more than 70% of physicians prescribe Kampo drugs today [7]. The Japan Society for Oriental Medicine is the biggest society for Kampo medicine and has 8600 members and 2600 certified board members. In 2001, Kampo education for medical students was incorporated into ‘the model core curriculum' by the Japanese Ministry of Education, Culture, Sports, Science and Technology [6].

The development of modern ready-to-use forms was directly related to the enormous increase in Kampo usage, mainly as spray-dried granular extracts of the original formulae. They have increasingly replaced the traditional decoction of the crude drugs, even though they are also covered by the national insurance system. Besides being simple to administer, industrial production has enabled several other advantages. The quality control of the purity as well as toxicity is standardized in Japan, following the Japanese pharmacopoeia and internationally established regulations for Good Manufacturing Practice (GMP) and Good Laboratory Practice (GLP). The standardization of the main components has become possible and this is a precondition of clinical research. Today, extract preparations make up to 95% of the Japanese Kampo market.

In Western countries, herbal therapies originating in other cultural areas, mainly Chinese herbal medicine as part of TCM, are receiving increasing interest. In the USA, TCM is still far more visible than Kampo. The practitioners practice herbal therapy often in combination with acupuncture, which is often a mixture of Chinese, Japanese and Korean acupuncture styles. Kampo drugs are only available over the counter, meeting Japanese GMP criteria. Since Japanese pharmaceutical companies have started clinical trials in the USA, several drugs have already been registered as investigational new drugs (IND) by the Food and Drug Administration. Safety and toxicity data from Japan are generally accepted by the US and European agencies.

In Europe, especially in Germany, there is a long-term tradition of herbal medicine, and there is growing interest in Chinese phytotherapy and Japanese Kampo is also getting more and more attention. However, there is a shortage of doctors specialized in Japanese Kampo.

### 1.3. Background of Kampo

Kampo is an individualized treatment system where the overall condition of the patient and their constitution are of real importance; additionally, Kampo has a holistic therapeutic approach, as the mind and body are seen as one entity. The therapeutic aim is to relieve symptoms and to restore harmony in bodily functions. The treatment regime is based on symptoms. For the determination of the appropriate herbal prescription, the physician carries out a thorough investigation of the complaints and symptoms of the patient, including taking their temperature, examining sensation, weakness or sweating, symptoms which are not often primarily taken into account in conventional medicine. The physical examination includes abdominal palpation, tongue inspection and pulse diagnosis. This provides additional information concerning the state of the disease, by gathering the amount and distribution of *ki* (vital energy), *ketsu* (blood) and *sui* (body fluid). The subjective complaints and the symptoms observed by the physician are combined to an individual symptom profile, a Kampo diagnosis (*sho*), which leads to the selection of the appropriate prescription [8]. It may happen that patients with the same conventional diagnosis obtain different prescriptions (same diagnosis but different treatments), or patients with different conventional diagnoses are prescribed the same formula (same treatment for different diagnoses).

Japanese physicians with limited education in Kampo diagnostics tend to apply the formulations according to conventional Western diagnoses. This makes sense for some limited indications, if the formula for the Kampo *sho* is close to the conventional diagnosis. However, in most cases, the traditional individual approach, where each patient receives their appropriate prescription, is the preferred option. For example, diseases that are expected to respond to the formula *Kakkonto* are diagnosed as *Kakkonto-sho* and it naturally follows that *Kakkonto* is prescribed in such cases.

These special conditions have made clinical research in the field of Kampo medicine more complex than the research on conventional drugs. The World Health Organization West Pacific Regional Office (WHO/WPRO) has put considerable efforts into standardizing East Asian traditional medicine [9]. WHO headquarters is considering incorporating the international classification of Traditional Medicine, East Asia (ICTM EA) into International Classification of Diseases (ICD)-11. ICD-11 is planned to be finalized in 2014 and scheduled to be approved by the WHO assembly in 2015. Japan proposes a double coding system of the ICD codes, that is, the conventional diagnosis code together with the traditional diagnosis (or pattern) code. This will allow integration into the conventional medical system without loosing the traditional information. The Kampo pattern (*sho*) codes have already been published in Japanese [10].

## 2. Information Available on Clinical Research

Our search was based on an evidence report of Kampo treatment made by the Japanese Society of Oriental Medicine (JSOM) which included 320 clinical trials between 1986 and 2008. [11]. This report includes Kampo trials available in the Cochrane register [12], ICHUSHI (Japan Medical Abstracts Society) [13] and the database from the Japan Kampo Medicines Manufacturer Association [14]. In this review only those studies were included, which used granulate formulations and were based on the drug regulation that was introduced in 1986. Liquid formulations and decoctions were excluded. Only peer-reviewed research from the JSOM database were included, which were also available in PubMed [15] or ICHUSHI [13]. A total of 135 trials, published between 1988 and 2007, were identified and summarized Table 1. These publications were extracted by two researchers fluent in both English and Japanese. Subsequently, they were discussed with two senior researchers (a Kampo specialist from Japan and a research methodologist from Germany). We classified Kampo clinical studies into three categories (Tables 2, 3, and 4):

- Kampo compared with either no treatment or different Kampo formula.
- Kampo compared with placebo.
- Kampo compared with standard treatment.

Among the 135 clinical studies, 106 were randomized controlled trials (RCTs), 6 quasi-RCTs and 10 were cross-over studies. There were 13 non-randomized comparative studies. Among the 106 RCTs, 23 studies were placebo-controlled. More than two thirds of the studies used only Kampo as verum, whereas in 38 studies, Kampo was used in addition to the standard treatment. Almost half of the studies (*n* = 68) used a standard control, 28 used a placebo control, 24 had no treatment control and in 15, another Kampo formula was used as a control Table 1.

The sample size varied between 4 patients in the smallest study and 2069 patients in the largest. Most of the studies were small. Two thirds included less than 100 patients and the overall quality was low. Thirty-five trials were published in English and the remaining studies were in Japanese. The spectrum of diagnoses was diverse. The most common diagnosis was asthma (ICD J 45.0 and J 45.9), which was evaluated in nine studies. Many of the trials had low methodological quality (small sample size and unclear concealment) and thus a publication bias is to be expected. With respect to the methodology, it is interesting to note that in all studies summarized here, the treatment was based on the Western diagnosis only. A Kampo diagnosis was not mentioned in any of the trials. However, one trial seemed to be more individually based, using seven different Kampo formula in the verum group [134].

## 3. Suggestions for Future Research

### 3.1. Relevant Research Questions

The research available followed a Western approach and concentrated on single Western diagnoses treated with one Kampo formula. Since Kampo is a comprehensive and complex treatment system with a traditional approach, many research questions still need to be investigated. Kampo has been used for hundreds of years and is well integrated into the Japanese health care system, therefore it should be taken into account using an appropriate research strategy. When performing clinical research identical questions must be addressed for every new treatment, as well as for traditional treatments, which are already on the market:

- For whom and what is it used to treat?
- Is it safe?
- Is it superior to placebo?
- Is it superior or equivalent to conventional standard treatment?

For traditional treatments, the order of research questions should differ from conventional drug research, because traditional treatments are already widely available [150]. First, knowledge is needed regarding who will benefit from the treatment and which diseases the treatment is intended to treat, as well as how it is to be administered. In addition, it would be helpful to get an idea as to whether the patients improve under the treatment not to mention the essential safety assessment.

All of these questions could be answered using a prospective observational study which evaluates these aspects in usual care. This has been done for other traditional treatments such as homeopathy [151–153], and is currently being carried out for Kampo at the Keio University [154]. This computer-based self-assessment system is divided into two domains. One is the patients' self-assessment at every visit using a visual analogue scale (VAS) and the other domain is an assessment by the physician. Data from both sources are combined and analyzed using data mining. The advantage of this system is that data is collected in a real-life setting. Also, Kampo values subjective complaints. This computer-based, self-assessment system, allows data incorporation of patient's subjective outcome measures.

Objective outcome measures which are often used in experimental RCTs are sometimes separated from subjective feelings. Kampo physicians value subjective complaints and diagnose *sho* not only based on objective findings, but also from the subjective complaints. The evaluation of the Kampo treatment by the physician is sometimes decided based on the subjective symptoms. Current Kampo clinical research has not taken this aspect into account. Due to the individualized treatment approach of Kampo, subjective outcome measures are relevant and should always be considered while planning a study. In addition to databases, some authors have also suggested that more individual single-case research including *N* of one trials would be suitable to reflect Kampo medicine [155, 156]. The main motivation to perform clinical research on traditional treatments is for justification purposes, most placebo-controlled trials on Kampo do not reflect the use of Kampo in usual practice and are therefore not helpful when making medical decisions in daily practice.

### 3.2. Testing for Superiority over Placebo

Previous research has followed the principles of conventional drug research by testing the superiority of a single Kampo formula over a placebo for a clearly defined conventional diagnosis. An RCT from 1998, for example, compared Rikkunshito with a kind of placebo (low dose of the same formula) for the treatment of dyspepsia [68]. Results from this kind of trial are helpful for the integration of a single formula into conventional care. A formula that has proven efficacy in a conventional drug trial could be used in the future without any Kampo knowledge. However, this provides no information about Kampo as a whole treatment system.

Nevertheless, this kind of research does not represent the traditional Kampo treatment. A traditional treatment is led from the Kampo diagnosis (by taking a patient history, abdominal examination, tongue and pulse diagnosis). If the aim of a clinical trial is to ask whether Kampo treatment in a traditional way is efficacious or not, the traditional treatment system has to be taken into account. For this purpose, an additional Kampo diagnosis with the conventional diagnosis could be used for choosing the appropriate Kampo medicine. There are two options; the first excludes the influences of the Kampo diagnostic procedure and the study could be performed in a similar way as suggested for the placebo-controlled study. When this design is used, the Kampo diagnosis is performed for all patients before randomization, although it is only needed for the group that actually receives Kampo.

**Figure 1:**
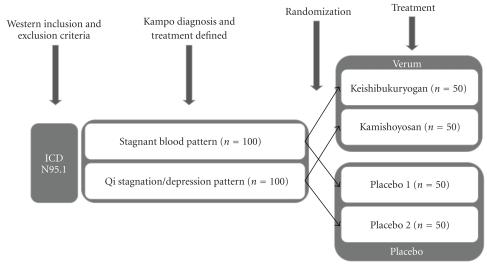
Combining the Western and Kampo diagnosis for a placebo-controlled trial: to evaluate the efficacy of Kampo drug treatment, for example, for menopausal symptoms (ICD N95.1).

The first design is that Kampo diagnoses and an appropriate treatment could be used for stratification within the randomization process (see Figure 1). This design is especially useful for pilot trials or smaller studies to prove Kampo as an individualized treatment system. This trial design allows for an individual Kampo treatment according to the Kampo diagnosis. However, it also means that a range of different formulae will be administered. In order to ensure blinding, it might be necessary to prepare an adequate placebo for each formula, if they differ in appearance, smell and taste. In this type of trial, the patients should not only be blinded for the treatment, but they should also not receive any information about their Kampo diagnosis. Designs like this have already been used for homeopathy [157].

For many Western diagnoses, more than two Kampo diagnoses are common. Different patterns would result in a larger number of subgroups and some of these might be too small to have enough statistical power for subgroup analysis. For this reason, it makes sense to use the pooled patterns for primary analysis and to pre-specify subgroup analysis for the more common patterns. Another possibility, which might be easier to handle for the trial process, is to use the Kampo diagnosis as additional inclusion criteria and to recruit only those patients with relevant Kampo diagnosis for the formula under research. An example for this can be seen in the study by Kobayashi [158]. When using this design, it must be recognized that a large number of patients may need to be screened. In addition, the results are less representative for the Western diagnosis and integration into conventional care might be more difficult, because Western trained doctors could not differentiate between the different Kampo diagnoses.

### 3.3. Testing for Non-Inferiority or Superiority over Standard Care

Doctors and patients want to know whether it is better to use Kampo instead of, or in addition to conventional treatment. Depending on its causality or external validity, the main focus of these studies could be performed more experimentally (homogenous patients and clearly defined treatment protocols) or more pragmatically (heterogeneous patients and a treatment which represents usual care) [159]. Especially for chronic diseases where a more complex treatment is needed [160], a pragmatic study design to test Kampo as an additional treatment could provide useful information for decision making. Similarly for the suggested placebo-controlled study designs, it is possible to use an individualized Kampo treatment within these studies.

**Figure 2:**
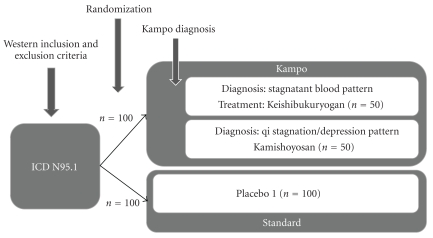
Combining the Western and Kampo diagnosis for a standard care controlled trial: to evaluate the effectiveness of Kampo as a whole treatment system, for example, for menopausal symptoms (ICD N95.1).

The second possibility is to see the diagnostic procedure as part of the Kampo treatment, and the diagnostic procedure be used only on the Kampo group after randomization Figure 2. Study designs shown in Figures 1 and 2 are used to answer different research questions. The first option focuses on the treatment effects of the drug, whereas the second option provides a broader picture and evaluates Kampo as a whole treatment system, which consists of both the diagnostic procedure and the drug treatment. 

### 3.4. Taking Patient Expectations into Account

Patient preferences and patient expectation can play a role in complementary and alternative medicine trials. Two systematic reviews suggest that the influence of patient expectations on outcomes is related to both within-group changes and between-group differences [161, 162]. This has already been shown for acupuncture [163]. If the patients in a Kampo trial have higher expectations of a positive outcome than the “average” patient, then this could result in within-group changes that are larger than in a more representative sample. High expectations might also be associated with high response rates and improved outcomes in the placebo-controlled group. This could result in a ceiling effect making it more difficult to detect a significant difference between verum and placebo. Different strategies are available to deal with this problem, such as: including a run-in phase, stratification for randomization and measuring expectation. A simple tool for measuring aspects of expectations at baseline is to ask questions such as: “How effective do you expect the treatment to be?” with responses such as “very effective”, “effective”, “slightly effective”, “not effective” or “don't know”. These data could be used to make adjustments in the primary data analysis.

## 4. Conclusion

Kampo is a holistic and individualized treatment with a long tradition and future research is required to take this into account. RCT is the appropriate study design for testing efficacy or effectiveness, however within such a study, the treatment should be individualized according to the Kampo diagnosis.

## Funding

This work was supported by Grant-in-Aide for Research on Applied Use of Statistics and Information, Health and Labour Sciences Research and Clinical Research for Development of Preventive Medicine and New Therapeutics from Ministry of Health, Labour and Welfare of Japan. This work was also supported by the Center for Clinical Trials, Japan Medical Association. Claudia Witts' Chair for Complementary Medicine is endowed by the Carstens-Foundation. Research grant for doctoral candidates from the German Academic Exchange Service (DAAD) to L. H.

## Figures and Tables

**Table 1:** Summary of Kampo clinical studies (1987–2007).

	RCT	Quasi-RCT	Cross-over design	Comparative study (non-randomized)	Total
Kampo versus either no treatment or a different Kampo formula	31	1	4	3	39
Kampo versus placebo	22	0	2	4	28
Kampo versus standard treatment	53	5	4	6	68

Total	106	6	10	13	135

**Table 2:** Kampo clinical studies comparing Kampo either with no treatment or with a different Kampo formula.

Author (year)	Reference	Language	ICD 10 code (disease name)	Design	*N*	Randomization	Blinding	Groups (*N*)	Intervention	Control	Kampo treatment
Seki et al. (1999)	[16]	J	A49.0 (MRSA colonizaton infection)	RCT	95	E	No	2	K	NT	hochuekkito
Konno et al. (1997)	[17]	J	C16.9 (gastric cancer)	RCT	23	E	No	2	SK	NT	juzentaihoto
Saito et al. (2006)	[18]	J	C80 (gastroenteric cancer)	RCT	48	E	No	2	K	NT	hochuekkito
Suzuki et al. (1995)	[19]	J	C80 (leukopenia with chemotherapy)	RCT	90	E	No	2	SK	NT	juzentaihoto
Higuchi et al. (2002)	[20]	J	D37.6 (liver ciirhosis)	RCT	52	E	No	2	K	NT	juzentaihoto
Ushiroyama et al. (2001)	[21]	E	E22.9 (endocrine fuction)	RCT	100	E	No	2	K	NT	unkeito
Ushiroyama et al. (2006)	[22]	E	E28.2 (polycystic ovary syndrome)	RCT	64	E	No	2	K	K	unkeito
Ushiroyama et al. (2003)	[23]	E	E28.3 (primary ovarian failure)	RCT	197	E	No	2	K	NT	unkeito
Namiki (2007)	[24]	J	E66.9 (obesity)	RCT	55	E	No	2	SK	NT	bofutsushosan
Iwasaki et al. (2005)	[25]	E	F05.1 (delirium superimposed on dementia)	RCT	52	R	No	2	K	NT	yokukansan
Aizawa et al. (2002)	[26]	E	F51.0 (nonorganic insomnia)	RCT-cross over	20	R	Yes	2	K	K	yokukansankachinpi-hange, anchusan
Higuchi et al. (2007)	[27]	E	G30.9 (alzheimer's disease)	RCT	75	R	No	3	K	NT	kihito, goshajinkigan
Ikeda et al. (2002)	[28]	E	H25.9 (senile cataract)	RCT	27	R	No	2	K	K	Kakkonto, saireito
Ikeda (2001)	[29]	E	H25.9 (senile cataract)	RCT	54	R	No	4	K	NT	orengedokuto, kakkonto, saireito
Abe (2002)	[30]	J	I89.0 (lymphoedema)	RCT	80	R	No	2	K	NT	goshajinkigan
Yoshimoto et al. (2002)	[31]	J	J30.1 (allergic rhinitis due to pollen)	Case Control	66	no	No	2	K	K	maobushisaishinto, shoseiryuto
Mori et al. (1999)	[32]	J	J30.1 (allergic rhinitis due to pollen)	Case Control	88	no	No	2	K	K	shoseiryuto, keimakakuhanto
Nishizawa et al. (2003)	[33]	J	J45.0 (predominantly allergic asthma)	RCT	139	R	DB	2	K	K	saibokuto, shoseiryuto
Mizuno et al. (2001)	[34]	J	K21.0 (reflux oesophagitis)	RCT	46	R	No	2	K	NT	rikkunshito
Nishida (2006)	[35]	J	K30 (dyspepsia)	RCT-cross over	11	no	No	2	K	K	rikkunshito
Oyabu et al. (1995)	[36]	J	K56.5 (intestinal adhesions)	RCT	53	E	No	2	K	NT	daikenchuto
Mori et al. (2003)	[37]	E	K59.1 (functional diarrhoea)	RCT	41	E	No	2	K	NT	hangeshashinto
Okabayashi et al. (1998)	[38]	J	K83.1 (obstruction of bile duct)	RCT	24	E	SB	2	SK	NT	inchinkoto
Endo et al. (2006)	[39]	E	K91.9 (postprocedural disorder)	RCT-cross over	17	no	No	2	SK	K	daikenchuto
Ohno (2006)	[40]	J	M35.0 (sicca syndrome)	quasi-RCT	64	no	No	2	K	K	bakumondoto, rokumigan, hachimijiogan, hochuekkito
Maejima et al. (2004)	[41]	J	M48.02 (spine deformity)	RCT	24	R	No	3	K	K	hachimijiogan, goshajinkigan,shuchibushimatsu
Yoshikawa et al. (1997)	[42]	J	N02.8 (childhood IgA nephropathy)	RCT	101	E	No	2	K	NT	saireito
Yoshikawa et al. (1998)	[43]	J	N04.9 (nephrotic syndrome)	RCT	171	E	No	2	SK	NT	saireito
Oribe et al. (2006)	[44]	J	N81.4 (postoperative discomfort for uterine prolapse)	RCT	19	No	No	2	K	NT	hachimijiogan
Takamatsu et al. (2002)	[45]	J	N95.8 (climacteric disorders)	Case Control	67	No	No	2	K	K	tokishakuyakusan, keishibukuryogan, kamishoyosan, juzentaihoto
Kawakami et al. (2003)	[46]	J	O92.5 (feeling of lactation deficiency)	RCT	72	R	No	6	K	K	kakkonto, juzentaihoto, kyukichoketsuin
Ushiroyama et al. (2005)	[47]	E	O99.3 (maternity blues)	RCT	268	E	No	2	K	NT	kyukichoketsuin
Yoshida (2000)	[48]	J	R11 (vomiting in children)	RCT	34	R	DB	2	K	K	goreisan and hochuekkito suppositorium
Nishizawa et al. (2000)	[49]	J	R25.2 (cirrhosis)	RCT	75	R	No	2	K	K	shakuyakukanzoto, goshajinkigan
Yoshikawa et al. (1997)	[50]	J	R31 (essential microscopic hematuria)	RCT	68	R	No	3	K	NT	kyukikyogaito, saireito
Kishida et al. (2007)	[51]	E	R60.9 (postoperative edema and inflammation)	RCT	17	R	No	2	K	NT	saireito
Hasegawa et al. (2002)	[52]	J	T45.1 (paclitaxel-induced myalgia)	RCT-cross over	15	R	No	2	SK	K	shakuyakukanzoto
Ueda et al. (1999)	[53]	J	Z22.8 (MRSA)	RCT	22	R	No	2	K	NT	Hochuekkito
Okawa et al. (1995)	[52]	J	Z51.0, D70 (leucopenia with radiotherapy of malignancies)	RCT	126	R	No	2	K	NT	ninjinyoeito

J: Japanese; E: English; R: randomization; E: envelops; DB: double blind; SB: single blind; K: Kampo; SK: Standard + Kampo; S: Standard; NT: no treatment; P: placebo; ICD: International Classification of Diseases, Kampo treatment includes only formulae produced after 1986. ICD codes details http://apps.who.int/classifications/apps/icd/icd10online/, dosage of the Kampo formulae http://www.jsom.or.jp/medical/ebm/index.html.

**Table 3:** Kampo clinical studies comparing Kampo with placebo.

Author (Year) (*N*)	Reference	Language	ICD 10 code (disease name)	Design	Cases (*N*)	Randomization	Blinding	Groups (*N*)	Intervention	Control	Kampo treatment
Suzuki et al. (2002)	[54]	J	A49.0 (MRSA)	RCT	13	R	DB	2	K	P	hochuekkito
Hioki et al. (2004)	[55]	E	E66.9 (obesity)	RCT	81	R	DB	2	K	P	bofutsushosan
Suzuki et al.(2005)	[56]	E	F03 (dementia)	RCT	30	R	DB	3	K	P	goshajinkigan, chotosan
Iwasaki et al. (2004)	[57]	E	F03 (dementia)	RCT	33	R	DB	2	K	P	hachimijiogan
Nagaki et al. (2003)	[58]	E	H16.1 (keratitis)	RCT	75	R	DB	3	K	P	goshajinkigan
Arakawa et al. (2006)	[59]	E	I10 (essential hypertension)	RCT	204	R	DB	2	K	P	orengedokuto
Nakamura et al. (2000)	[60]	J	I95.1 (Orthostatic hypotension)	RCT-cross over	10	R	SB	2	K	P	goreisan
Kaji et al. (2001)	[61]	J	J00 (acute nasopharyngitis)	RCT	250	R	DB	2	K	P	shosaikoto
Baba (1995)	[62]	J	J30.4 (allergic rhinitis)	RCT	217	E	DB	2	K	P	shoseiryuto
Miyamoto et al. (2001)	[63]	J	J40 (bronchitis)	RCT	192	R	DB	2	K	P	shoseiryuto
Urata et al. (2002)	[64]	E	J45.0 (bronchial asthma)	RCT-cross over	33	R	DB	2	K	P	saibokuto
Nishizawa et al. (2001)	[65]	J	J45.0 (bronchial asthma)	RCT	32	R	DB	2	K	P	Saibokuto inhalation
Nishizawa et al. (2001)	[66]	J	J45.0 (bronchial asthma)	RCT	74	R	DB	2	K	P	Saibokuto inhalation
Iwasaki et al. (2007)	[67]	E	J69.0 (pneumonitis)	RCT	95	R	DB	2	K	P	hangenkoubokuto
Harasawa et al. (1998)	[68]	J	K31.9 (dysmotility-like dyspepsia)	RCT	296	R	DB	2	K	P	rikkunshito
Sasaki et al. (1998)	[69]	J	K58 (irritable bowel syndrome)	RCT	204	E	DB	2	K	P	keishikashakuyakuto
Miyoshi et al. (1994)	[70]	J	K59.0 (constipation)	RCT	146	E	DB	3	K	P	daiokanzoto
Itoh et al. (2002)	[71]	E	K91.3 (post-operative ileus)	RCT	24	R	SB	2	K	P	daikenchuto
Takagaki et al. (2000)	[72]	J	K91.3 (paralytic ileus)	RCT	21	R	SB	2	K	P	daikenchuto
Nishizawa et al. (2004)	[73]	E/J	M35.0 (Sicca syndrome)	RCT	229	R	DB	2	K	P	bakumondoto
Aoki et al. (2001)	[74]	E	N39.9 (urodynamic studies)	RCT-cross over	19	R	SB	2	K	P	maobushisaishinto
Kumada et al. (1999)	[75]	J	R25.2 (muscle cramps)	RCT	126	R	DB	2	K	P	shakuyakukanzoto
Odaguchi et al. (2006)	[76]	E/J	R51 (headache)	RCT	53	R	DB	2	K	P	goshuyuto
Satoh et al. (2005)	[77]	E	R54 (senile muscle weekness)	RCT	13	R	DB	3	K	P	hochuekkito
Hamazaki et al. (2007)	[78]	E	Z01.8 (adjuvant effect to vaccination)	RCT	36	R	DB	2	K	P	hochuekkito
Takahashi et al. (2007)	[79]	E	Z01.8 (serum amino acid concentration)	RCT-cross over	18	R	SB	3	K	P	rokumigan
Saruwatari et al. (2004)	[80]	E	Z01.8 (COPD)	RCT-cross over	26	R	DB	2	K	P	bakumondoto
Isobe et al. (2003)	[81]	E	Z01.9 (retinal blood flow)	RCT-cross over	12	R	DB	2	K	P	hachimijiogan

J: Japanese; E: English; R: randomization; E: envelops; DB: double blind; SB: single blind; K: Kampo; SK: Standard + Kampo; S: Standard; NT: no treatment; P: placebo; ICD: International Classification of Diseases, Kampo treatment includes only formulae produced after 1986, ICD codes details http://apps.who.int/classifications/apps/icd/icd10online/, dosage of the Kampo formulae http://www.jsom.or.jp/medical/ebm/index.html.

**Table 4:** Kampo clinical studies comparing Kampo with standard treatment.

Author (Year)	Reference	Language	ICD 10 (disease)	Design	Cases (*N*)	Randomization	Blinding	Groups (*N*)	Intervention	Control	Kampo treatment
Sasaki et al. (2006)	[82]	J	C18.9 (gastroenteric cancer)	RCT	168	No	No	2	SK	S, K	juzentaihoto
Sasaki et al. (2007)	[83]	J	C18.9 (cancer chemotherapy)	RCT	168	No	No	2	SK	S, K	juzentaihoto
Adachi (1988)	[84]	J	C50.9 (advanced breast cancer)	RCT	74	E	No	2	SK	S	juzentaihoto
Yamamoto et al. (2003)	[85]	J	D25.9 (uterine adenomyosis)	RCT	24	R	No	2	SK	S	keishibukuryogan
Akase et al. (2003)	[86]	J	D50.0 (anemia due to uterine myoma)	RCT	25	R	No	2	K	S	tokishakuyakusan
Aoe (2007)	[87]	J	D50.8 (iron deficiency anemia)	RCT	120	R	No	3	SK	S	juzentaihoto
Yanagibori et al. (1995)	[88]	J	D50.9 (Iron deficiency anemia,)	RCT	39	E	SB	2	SK	S	ninjinyoeito
Aoe et al. (2000)	[89]	J	D62 (acute posthaemorrhagic anemia)	RCT	57	R	No	2	SK	S	juzentaihoto
Aoe et al. (1999)	[90]	J	D62 (acute posthaemorrhagic anemia)	RCT	90	R	No	3	SK	S	juzentaihoto, ninjinyoeito
Azuma et al. (1994)	[91]	J	E10-E14 (non-insulin-dependent diabetes mellitus)	RCT	18	E	No	2	SK	S	seishinrenshiin
Yamano et al. (1995)	[92]	J	E78.5 (hyperlipidaemia)	RCT	92	E	No	3	SK	S, NT	daisaikoto
Sasaki et al. (1991)	[93]	J	E78.5 (hyperlipidaemia)	RCT	40	R	No	3	SK	S	daisaikoto
Ishida et al. (1999)	[94]	J	F41.9 (anxiety disorder, unspecified)	RCT	15	R	No	2	SK	S	saibokuto
Yamagiwa and Fujita (2007)	[95]	J	F45.3 (abnomal sensation)	quasi-RCT	86	R	No	2	K	S	rikkunshito
Maruyama (2006)	[96]	J	G43.9 (migraine, unspecified)	RCT-cross over	28	R	No	2	K	S	goshuyuto
Kimura et al. (1991)	[97]	J	G51.3 (facial spasm)	RCT	20	R	No	2	SK	S	shakuyakukanzoto
Sekine et al. (2003)	[98]	J	G54.4 (lumbosacral root disorders)	RCT-cross over	20	R	No	2	K	S	goshajinkigan
Inoue (2001)	[99]	J	H65.0 (acute serous otitis media)	Case Control	34	No	No	2	K	S	shoseiryuto, eppikajutsuto
Matsushita et al. (1995)	[100]	J	I67.9, I67.8, I10 (cerebrovascular disease, hypertension)	RCT	22	E	No	2	K	S	chotosn
Akiyama et al. (2001)	[101]	J	I73.0 (raynaud's syndrome)	Case Control	49	No	No	2	SK	S	tokishakuyakusan, orengedokuto
Fujimori et al. (2001)	[102]	J	J00 (postinfections cough)	RCT	25	R	No	2	K	S	bakumondoto
Kimoto and Kuroki (2005)	[103]	J	J10 (influenza)	Case Control	19	No	No	2	SK	S	maoto
Kubo et al. (2007)	[104]	E	J10.1 (type A influenza infection)	quasi-RCT	37	No	No	2	SK	S	maoto
Kato et al. (2005)	[105]	J	J44.9 (chronic obstructive pulmonary disease)	RCT	31	E	No	2	SK	S	seihaito
Tatsumi et al. (2009)	[106]	E	J44.9 (chronic obstructive pulmonary disease)	RCT	71	E	No	2	K	S	hochuekkito
Nishizawa et al. (2004)	[107]	J	J45.0 (bronchial asthma)	RCT	161	R	No	2	K	S	shinbito inhalation
Nishizawa et al. (2003)	[108]	J	J45.0 (bronchial asthma)	RCT	114	R	No	2	K	S	shinbito inhalation
Nishizawa et al. (2002)	[109]	J	J45.0 (bronchial asthma)	RCT	107	R	No	2	K	S	saibokuto
Nishizawa et al. (2002)	[110]	J	J45.0 (bronchial asthma)	RCT	94	R	No	2	K	S	saibokuto inhalation
Egashira and Nagano (1993)	[111]	E	J45.9 (bronchial asthma)	RCT	112	E	SB	2	SK	S	saibokuto
Mikamo et al. (2007)	[112]	J	J98.9 (respiratory infection)	RCT	116	No	No	3	SK	S	jumihaidokuto, kakkoto, keishito, kososan, shosaikoto, hochuekkito
Umemoto et al. (2007)	[113]	J	K11.7 (dry mouth)	RCT	100	No	No	3	K	S	bakumondoto
Yamada et al. (1998)	[114]	J	K14.6 (glossodynia)	RCT	104	R	No	2	K	S	saibokuto
Kato et al. (2005)	[115]	J	K21.0 (gastro-oesophageal reflux disease with oesophagitis)	RCT	19	E	No	2	SK	S	hangekobokuto
Koide (2006)	[116]	J	K21.9 (gastro-oesophageal reflux disease without oesophagitis)	RCT	118	No	No	3	SK, K	S, K	rikkunshito
Higuchi et al. (1999)	[117]	E	K26.9 (Helicobacter pylori)	RCT	63	R	No	2	SK	S	goshuyuto
Yamaguchi and Koide (2007)	[118]	J	K30 (dyspepsia)	RCT	120	E	No	3	K	S	rikkunshito
Nishizawa et al. (2004)	[119]	J	K59.0 (constipation)	RCT	318	R	No	2	SK	S	kumibinroto
Nakajima et al. (2003)	[120]	J	K73.9 (chronic hepatitis)	RCT	100	E	No	3	K	S, K	shosaikoto
Nakajima et al. (1999)	[121]	J	K73.9 (chronic hepatitis)	RCT	99	R	No	2	SK	S	shosaikoto
Tarao (2007)	[122]	J	K73.9 (chronic hepatitis)	RCT	156	No	No	2	K	K	shosaikoto, juzentaihoto
Okuma (1993)	[123]	J	L70.0 (cne vulgaris)	RCT	268	R	No	5	K	S, K	jumihaidokuto, orengedokuto
Nishizawa et al. (2004)	[124]	J	M35.0 (sicca syndrome)	RCT	847	R	No	2	K	S	bakumondoto
Nishizawa et al. (2003)	[125]	J	M35.0 (Sicca syndrome)	RCT	756	R	No	2	K	S	bakumondoto
Nishizawa et al. (2002)	[126]	J	M35.0 (Sicca syndrome)	RCT	105	C	No	2	K	S	bakumondoto
Hayashi et al. (1994)	[127]	J	M48.0 (spinal stenosis)	Quasi-RCT	27	R	No	2	K	S	hachimijiogan
Maejima and Katayama (2004)	[128]	J	M48.02 (chronic lumbar pain)	RCT	89	R	No	3	K	S, K	goshajinkigan, shuchibushimatsu
Nishizawa et al. (2007)	[129]	J	N32.8 (overactive bladder)	RCT	704	No	No	2	K	S	goshajinkigan
Iwabuchi (2000)	[130]	J	N93.9 (dysfunctional uterine bleeding)	Case Control	183	No	No	2	K	S	kyukikyogaito
Takamatsu (2006)	[131]	J	N95.1 (menopausal syndrome)	Quasi-RCT	170	No	No	2	K	S	tokishakuyakusan, kamishoyosan keishibukuryogan
Ushiroyama et al. (2005)	[132]	E	N95.8 (menopausal syndrome)	RCT	131	R	No	2	K	S	keishibukuryogan
Matsuo et al. (2005)	[133]	J	N95.8 (menopausal syndrome)	RCT-cross over	24	R	No	2	SK	S	tokishakuyakusan
Ota et al. (2001)	[134]	J	N95.8 (menopausal syndrome)	RCT	96	R	No	2	K	S	keishibukuryogan, kamishoyosan,goshajinkigan, tokishakuyakusan, tokakujokito, kihito, nyoshinsan
Ushiroyama et al. (2006)	[135]	J	O03.9 (uterine hemorrhage)	RCT	72	R	No	2	K	S	kyukikyugaito
Wada et al. (2003)	[136]	J	O90.9 (postpartum condition)	RCT	60	R	No	2	K	S	kyukichoketsuin
Sakuma et al. (2002)	[137]	J	O90.9 (postpartum psycho-physical condition)	RCT	171	R	No	2	K	S	kyukichoketsuin
Takushima and Inoguchi (2001)	[138]	J	O90.9 (puerperium)	Case Control	47	No	No	2	K	S	kyukichoketsuin
Nishizawa et al. (2003)	[139]	J	R05 (cough)	RCT	2069	R	No	2	K	S	bakumondoto
Motoo et al. (2005)	[140]	E	T37.5 (ribavirin-induced anemia)	RCT	23	R	No	2	K	S	ninjinyoeito
Hushiki et al. (2003)	[141]	J	T45.4 (gastritis due to oral iron)	RCT	120	R	No	2	SK	S	rikkunshito
Imazato et al. (1998)	[142]	J	Z01.8 (pretreatment of barium enema)	RCT	60	R	No	2	SK	S	shakuyakukanzoto
Yokota et al. (1990)	[143]	J	Z01.8 (pretreatment of barium enema)	RCT	60	R	No	2	K	S	daiokanzoto
Saida et al. (2003)	[144]	J	Z01.8 (pretreatment for bowel endscopy)	RCT	70	E	No	2	SK	S	shakuyakukanzoto
Ohnishi et al. (1999)	[145]	E	Z01.9 (pharmacokinetics with carbamazepine)	RCT-cross over	4	R	No	2	SK	S	shoseiryuto
Saida et al. (2005)	[146]	E	Z03.1 (pretreatment of colonoscopy)	RCT	285	E	No	2	SK	S	daikenchuto
Sugihara (1999)	[147]	J	Z03.1 (gastric endoscopy)	Case Control	58	No	No	2	SK	S	shakuyakukanzoto
Mizukami et al. (2007)	[148]	J	Z03.8 (pretreatment of colonoscopy)	Quasi-RCT	42	No	No	2	SK	S	shakuyakukanzoto
Ai et al. (2006)	[149]	E	Z03.8 (pretreatment of colonoscopy)	RCT	110	R	No	2	K	S	shakuyakukanzoto

J: Japanese; E: English; R: randomization; E: envelops; DB: double blind; SB: single blind; K: Kampo; SK: Standard+Kampo; S: Standard; NT: no treatment; P: placebo; ICD: International Classification of Diseases, Kampo treatment includes only formulae produced after 1986, ICD codes details http://apps.who.int/classifications/apps/icd/icd10online/, dosage of the Kampo formulae http://www.jsom.or.jp/medical/ebm/index.html.
